# Analysis method for PCBs in reclaimed oil using a fast-GC triple stage quadrupole mass spectrometer with the 13-component quantitation method

**DOI:** 10.1007/s11356-017-0533-x

**Published:** 2017-12-04

**Authors:** Hiroshi Takakuwa, Takashi Miura, Toru Matsumura, Norie Hashi, Michiyo Kubota, Hiroshi Kutsukake, Kazuhiro Muramatsu, Takashi Kasamatsu, Masahiro Okuda

**Affiliations:** 1 Agilent Technologies Japan Ltd., 9-1 Takakura-Cho, Hachioji, Tokyo, 192-8510 Japan; 2 Kiraku Kogyo Co., Ltd., 2-7-33 Ishibeguchi, Konan, Shiga 520-3144 Japan; 3 IDEA Consultants, Inc., 1334-5 Riemon, Yaizu, Shizuoka, 421-0212 Japan; 4 EIS Japan Co., Ltd., 1334-5 Riemon, Yaizu, Shizuoka, 421-0212 Japan

**Keywords:** Polychlorinated biphenyls, Reclaimed oil, Fast-GC, Triple stage quadrupole mass spectrometer, Sample preparation

## Abstract

It is necessary for companies supplying reclaimed oil to analyze polychlorinated biphenyls (PCBs), because there is a possibility of the presence of contaminants due to trace-level PCBs in the reclaimed oil. However, common analysis methods of PCBs are time-consuming and complicated. Fast-GC triple stage quadrupole mass spectrometer with the 13-component quantitation method is an official method for analyzing PCBs in insulating oil in Japan. This method is extremely fast and simplified. The purpose of this study involves an investigation of the aforementioned fast and simple method for potential use in the analysis of reclaimed oil. Furthermore, it was attempted to combine the method with sample preparation involving only hexane dilution. The effect of sample dilutions corresponding to 100, 300, and 500 times was evaluated for reducing the matrix effect. The matrix effect was suppressed at a dilution ratio equal to or exceeding 300 times. Calibration curves of four points, namely 0.01, 0.05, 0.1, and 0.5 ng/mL, (ignored origin) by using an internal standard method were prepared for the 13 components. The square of regression coefficient (R^2^) values of all calibration curves exceeded 0.997. This method was adopted for the analysis of reclaimed oil containing 0.5 μg/mL PCBs, which corresponds to the judgment criteria, and accurate quantitation (accuracy value, 94.0–102%) and good repeatability (%RSD, 3.6%) were obtained. Furthermore, the required sensitivity was maintained even when 800 samples were analyzed without a cleaning ion source and an exchanging analysis column.

## Introduction

In the manufacture and supply of reclaimed oil, safety and good quality of the end products is important. Suppliers of reclaimed oils collect waste lubricating oils such as engine oil, gear oil, hydraulic oil, heavy oil, and insulating oil. as raw materials, and recycle these oils at their plants. The lubricating waste oil could potentially be contaminated with trace-level poly chlorinated biphenyls (PCBs). Therefore, it is necessary for companies supplying reclaimed oil to analyze PCBs in reclaimed oil for quality control. Furthermore, an important issue involves speeding up PCB analysis to increase the production efficiency of reclaimed oil. Many studies have developed methods for the analysis of PCBs in waste oil. However, the aforementioned methods are extremely time-consuming in terms of performing a series involving sample preparation, GC measurement, and data analysis. Specifically, methods including sulfuric acid treatment, partition with dimethyl sulfoxide (DMSO)/hexane, partition with dimethylformamide (DMF)/hexane, florisil column cleanup, and silica gel column cleanup are used in sample preparation, and the operations require skilled technologies in terms of analysis and operation time (Copland and Gohmann 1982; Orazio et al. 1989; Larsen et al. 1991; Lawn and Toffel 1987; Gordon et al. 1982; Sandra et al. 1988; Suzuki et al. 1991; Koizumi and Yoshimura 1984). Conversely, with respect to the analysis of insulation oil analysis in Japan, a few pretreatment techniques were developed to aid in performing the analysis in a more quick and easy manner (Takahashi and Honda 2010; Shimizu 2010; Hamada 2010). Furthermore, analytical methods by sample preparation with only hexane dilution were developed in gas chromatography combined with negative chemical ionization (GC/NCI) and gas chromatography combined with high resolution mass spectrometry (GC/HRMS) (Takasuga et al. 2006; Machii et al. 2003). In this study, we attempted to perform an analysis by using a gas chromatography combined with triple stage quadrupole mass spectrometer (GC/MS/MS) with high selectivity involving only hexane dilution. The GC/MS/MS was selected because of its simple operability and maintainability when compared with those of GC/NCI and GC/HRMS. Additionally, the detection limit of recent GC/MS/MS is equivalent to that of GC/HRMS due to technological innovations, and this is also adopted in the official method for the analysis of dioxins in food by the European Union (EC 589/2014 and EC 709/2014). The selection of the analytical column is an important factor that can provide a high speed in GC analysis. Extant studies examined several columns as columns for PCB analysis (Frame 1997), and this typically corresponds to DB-5MS and HT8-PCB in Japan (Matsumura et al. 2002). However, the analysis time approximately equals or exceeds 40 min when these columns are used. In contrast, studies reported on high-speed analytical methods involving the use of a VF-Rapid MS PCB screen column that elute all PCB congeners of three Arochlors within 4 min (Cochran 2002). The column corresponds to a wide bore column fitted with a resistance column with a length of 6 m and an inner diameter of 0.1 mm at the tip. This column was adopted in the present study. The analysis cycle time using this column approximately corresponds to 13.5 min including the oven cooling time. In the ordinary data analysis of PCBs, it is necessary to perform data processing on 209 congeners, and this constitutes a time-consuming process similar to that of pretreatment. Japan’s insulating oil manual presents a method for quantifying the total PCB using statistical analysis based on the quantitative values of 13 components and is available online at http://www.env.go.jp/press/files/jp/17471.pdf (Ministry of Environment May 11, 2011). In this method, since only 13 components are the target components, it is overwhelmingly convenient when compared with the ordinary method. This method is applicable to GC/MS, GC/HRMS, and GC/MS/MS, but cannot be used with NCI. In this study, we investigate the applicability of the fast-GC triple stage quadrupole mass spectrometer with 13-component quantitation method to the analysis of reclaimed oil.

## Materials and methods

### Regents, chemicals, and sample

Calibration solution of PCBs: EC-5488 and internal standard solutions; EC-5379, and EC-5450 were purchased from Cambridge Isotope Laboratories Inc. (MA, USA). The EC-5488 contains reference solutions of four concentrations, namely CS 1, CS 2, CS 3, and CS 4. The concentrations of these solutions are given in Table 1. The solutions were diluted with hexane to prepare 0.01, 0.05, 0.1, and 0.5 ng/mL unlabeled PCBs congeners with 0.1 ng/mL 13C-labeled PCB congeners. The four standard solutions were used to prepare 4-point internal standard calibration curves with “ignore origin.” The reclaimed oil sample in which PCBs were not detected was prepared by Kiraku Kogyo Co., Ltd. (Shiga, Japan). Kanechlor (KC) (300, 400, 500, and 600) and KC-Mix (KC-300: KC-400: KC-500: KC-600 = 1:1:1:1) for preparing reclaimed oil containing PCBs were purchased from GL Sciences Inc. (Tokyo, Japan).

**Table 1:** List of concentrations of EC-5488 (unit: ng/mL)

IUPAC#	CS1	CS2	CS3	CS4
Native				
18	1	5	10	50
28	1	5	10	50
44	1	5	10	50
52	1	5	10	50
70	1	5	10	50
101	1	5	10	50
110	1	5	10	50
118	1	5	10	50
138	1	5	10	50
149	1	5	10	50
153	1	5	10	50
180	1	5	10	50
187	1	5	10	50
Labeled				
28	10	10	10	10
52	10	10	10	10
70	10	10	10	10
101	10	10	10	10
118	10	10	10	10
138	10	10	10	10
141	10	10	10	10
153	10	10	10	10
180	10	10	10	10

### Sample preparation

Dilution of reclaimed oil with hexane was performed for the sample preparation. An evaluation was performed as to whether sample dilutions by 100, 300, and 500 times reduce the matrix effect. The diluted solutions were prepared by adding 1, 3, and 5 mL of 0.1 ng/mL internal solution to 0.010 g reclaimed oil. The evaluation was performed by comparing the diluted solutions to internal standard solutions for peak intensities as obtained by GC/MS/MS analysis. Specifically, 13C-labeled PCB congeners (#28, #52, #70, #101, #118, #138, #153, and #180) were used as the internal standard (IS) to correct the sensitivity of the peaks of the 13 components. The concentration of IS in the sample was adjusted to 0.1 ng/mL.

### GC/MS/MS analysis and quantification

Additionally, PCB analysis was performed by GC/MS/MS (7890B GC/7010B triple quadrupole MS Agilent Technologies, USA). The samples were analyzed with a VF-Rapid MS PCB screen column (Agilent Technologies, USA), which corresponds to a wide bore analysis column (10 m, 0.53 mm, 0.25 μm) combined with pre-restrictor (0.6 m, 0.1 mm) with the following temperature programs: 85 °C for 1 min—40 °C/min—305 °C for 3 min. The analysis time only corresponds to 13.5 min per sample including cooling oven time. The GC/MS/MS was operated in selected reaction monitoring (SRM). The SRM conditions are given in Table 2. Quantitation of total PCB concentration by using the 13-component quantitation method was conducted by using the PCBs calculation software provided by EIS Japan Co., Ltd. (Shizuoka, Japan). Table 3 shows the 13 components used for calculation and PCB congeners contained in each component. Table 4 shows the correspondence between the 13 target components and the 13C-labeled PCB congeners as internal standards.

**Table 2:** SRM conditions: precursor ion and product ion (unit: m/z) and collision energy (unit: eV)

Compounds	Precursor ion	Product ion	Collision energy
TrCB	257.8	186	34
TrCB	255.8	151	50
TrCB	255.8	186	32
TrCB^-13^C_12_	269.8	198	34
TrCB^-13^C_12_	267.8	163	50
TrCB^-13^C_12_	267.8	198	32
TeCB	291.8	222	34
TeCB	291.8	220	34
TeCB	291.8	257	12
TeCB^-13^C_12_	303.8	234	34
TeCB^-13^C_12_	303.8	232	34
TeCB^-13^C_12_	303.8	269	12
PeCB	325.9	256	34
PeCB	325.9	254	34
PeCB	325.9	291	12
PeCB^-13^C_12_	337.9	268	34
PeCB^-13^C_12_	337.9	266	34
PeCB^-13^C_12_	337.9	303	12
HxCB	361.7	290	30
HxCB	359.8	290	35
HxCB	359.8	325	15
HxCB^-13^C_12_	373.7	302	30
HxCB^-13^C_12_	371.8	302	35
HxCB^-13^C_12_	371.8	337	15
HpCB	395.8	325.9	30
HpCB	393.8	323.9	35
HpCB	391.8	321.9	30
HpCB^-13^C_12_	407.8	337.9	30
HpCB^-13^C_12_	405.8	335.9	35
HpCB^-13^C_12_	403.8	333.9	30

**Table 3:** Thirteen components used for calculation and PCB congeners contained in each component

Type of homologue	Component number	IUPAC number of major PCB congener						
TrCB	1	17	18					
	2	28	31					
TeCB	3	49	52					
	4	44						
	5	58	61	63	66	70	74	76
PeCB	6	89	90	101	113			
	7	85	110	120				
	8	107	118	123				
HxCB	9	139	140	147	149			
	10	132	153	168				
	11	130	138	158	160	163	164	
HpCB	12	175	182	183	187			
	13	172	180	191	193

**Table 4:** Table of correspondence between 13 target components and 13C-labeled PCB congeners as internal standards

Component1	Component2	Component3	Component4	Component5	Component6	Component7
#28	#28	#52	#52	#70	#101	#118
Component8	Component9	Component10	Component11	Component12	Component13	
#118	#153	#153	#138	#180	#180	

## Results and discussion

### Investigation of dilution rate

**Fig. 1:**
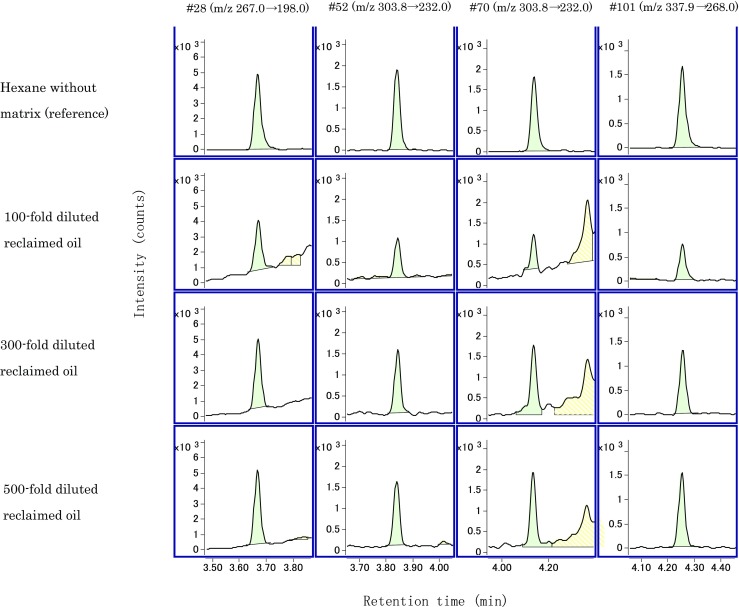
SRM chromatograms of 0.1 ng/mL internal standard substances in hexane (reference) and 100, 300, and 500 times diluted reclaimed oil (#28, 52, 70, 101)

**Fig. 2:**
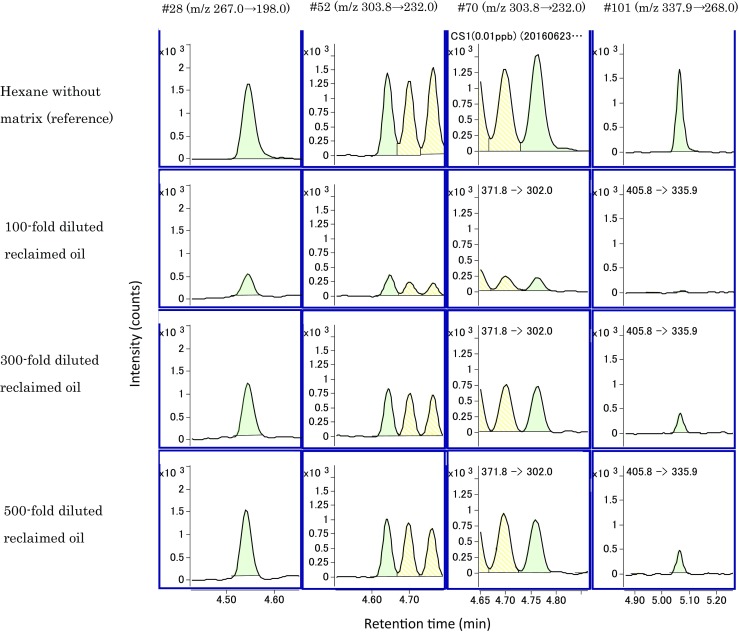
SRM chromatograms of 0.1 ng/mL internal standard substances in hexane (reference) and 100, 300, and 500 times diluted reclaimed oil (#118, 153, 138, 180)

Figure 1 and Figure 2 show the SRM chromatograms of 0.1 ng/mL internal standard substances in 100, 300, and 500 times diluted reclaimed oil relative to the chromatograph of 0.1 ng/mL internal standard substances in the hexane solution (reference). Table 5 shows each relative peak area value of the chromatograms when the peak area value of reference is 100. With respect to the 100 times diluted solution, the area values of the four congeners with fast retention time (#28, #52, #70, and #101) exceeded half of the reference while the area values of three congeners with slow retention time (#118, #153, and #138) corresponded to 17.1–33.7% of the reference. Furthermore, the area value of #180 was 4.76% of the reference, and this resulted in a large decrease in strength. Conversely, with respect to the conditions involving 300 and 500 times, the area values of all congeners excluding congener #180 exceeded half of the reference, namely 54.4–147%. Furthermore, the area values of #180 corresponded to 38 and 43.8%. Hence, the influence of the matrix was suppressed at a dilution ratio equal to or exceeding 300 times. Therefore, the dilution ratio was determined as 300 times. It was thought the fact that the values of #28, #52, #70, #101, and #118 exceeded 100% is attributed to a positive matrix effect. As widely-known, the matrix effects in GC/MS analysis are negative as well as positive due to the occurrence of ion enhancement (Coelho and Franco 2016). However, the 13C-labeled PCB congeners were used as congeners in the internal standard substance, and thus it is assumed that the ion enhancement can be corrected.

**Table 5:** Relative peak area values with respect to the peak area value of the hexane solution (reference) corresponding to 100

	#28	#52	#70	#101	#118	#153	#138	#180
100 diluted reclaimed oil	93.0	72.5	56.7	60.7	32.9	33.7	17.1	4.76
300 diluted reclaimed oil	115	115	145	104	80.6	72.0	54.4	38.0
500 diluted reclaimed oil	126	113	147	126	107	90.0	68.9	43.8

### Calibration curve

The 4-point (0.01, 0.05, 0.1, and 0.5 ng/mL) calibration curves with ignore origin by internal standard method were prepared for the 13 components. The 13 components at 0.01 ng/mL were detectable. The signal to noise (S/N) (peak to peak) values of each component corresponded to 4.1–22.1 (Table 6). The analytical curve equation and square of regression coefficient (R^2^) of the calibration curves are listed in Table 7. The R^2^ values of all calibration curves exceeded 0.997.

**Table 6:** Signal to noise ratio of the 13 components at 0.01 ng/mL and noise calculation range

	Component1	Component2	Component3	Component4	Component5	Component6	Component7
Signal to noise	22.1	16.0	6.5	6.0	6.0	4.1	5.0
Noise calculation range	2.90–3.00 min	2.90–3.00 min	3.45–3.55 min	3.45–3.55 min	3.45–3.55 min	3.50–3.60 min	3.50–3.60 min
	Component8	Component9	Component10	Component11	Component12	Component13	
Signal to noise	6.9	11.8	8.3	10.3	9.5	15.5	
Noise calculation range	3.50–3.60 min	3.95–4.05 min	3.95–4.05 min	3.95–4.05 min	4.00–4.10 min	4.00–4.10 min

**Table 7:** Analytical curve equation and the square of regression coefficient (R^2^)

	Analytical curve equation	R2
Component1 (TrCB)	*y* = 0.011258 *x* + 0.062471	0.999931
Component2 (TrCB)	*y* = 0.010924 *x* + 0.083801	0.999942
Component3 (TeCB)	*y* = 0.009558 *x* + 0.065022	0.999710
Component4 (TeCB)	*y* = 0.009907 *x* − 0.077633	0.997655
Component5 (TeCB)	*y* = 0.009053 *x* + 0.092591	0.999340
Component6 (PeCB)	*y* = 0.009509 *x* − 0.057573	0.999702
Component7 (PeCB)	*y* = 0.006831 *x* + 0.077679	0.999794
Component8 (PeCB)	*y* = 0.006565 *x* + 0.054137	0.999410
Component9 (HxCB)	*y* = 0.011756 *x* − 0.048442	0.998965
Component10 (HxCB)	*y* = 0.011713 *x* − 0.009537	0.999773
Component11 (HxCB)	*y* = 0.007932 *x* + 0.030521	0.999812
Component12 (HpCB)	*y* = 0.008407 *x* − 0.019222	0.999759
Component13 (HpCB)	*y* = 0.007983 *x* + 0.020156	0.999865

### Trueness of total PCBs concentration

**Fig. 3:**
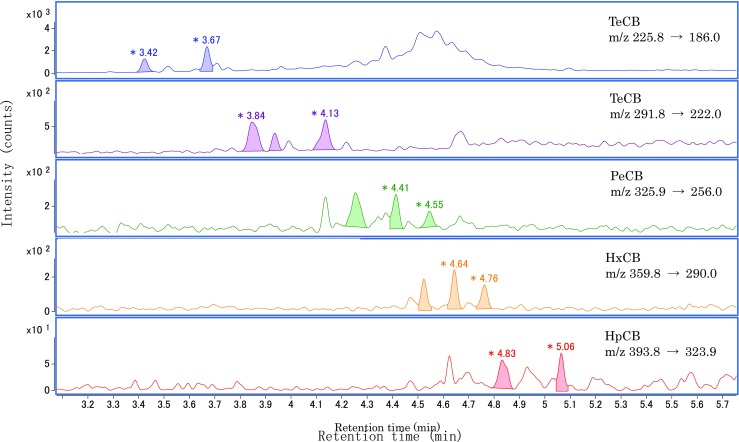
SRM chromatograms of the reclaimed oil containing 0.5 μg/mL KC-mix

**Fig. 4:**
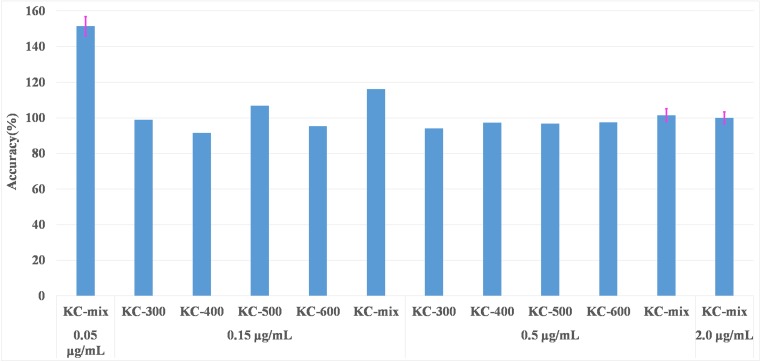
Bar charts with error bars of the accuracy of total PCB concentration in reclaimed oil samples containing Kanechlor standard

Japanese regulations specify that the maximum residue limit is 0.5 μg/mL and that the limit of detection (LOD) in analysis method is less than 0.15 μg/mL. Therefore, it is necessary to correctly quantify 0.5 μg/mL. Reclaimed oil containing 0.15 and 0.5 μg/mL Kanechlor standard was analyzed, and the total PCB concentration was calculated. Additionally, reclaimed oil containing lower and higher concentrations (0.05 and 2.0 μg/mL) were also analyzed. The samples of reclaimed oil containing 0.05, 0.5, and 2.0 μg/mL of the KC-mix were repeatedly analyzed five times. Accuracy is derived as follows: [The measured value]/[The true value] × 100 (%). The targeted 13 components were detected in all the reclaimed oil samples by adding the KC standard. Figure 3 shows the SRM chromatograms of the reclaimed oil containing 0.5 μg/mL KC-mix. Figure 4 shows the bar chart with error bars indicating the accuracy of the total PCB concentration. The accuracy values of 0.5 μg/mL were 94.0–102%. Additionally, the accuracy values of 0.15 and 2.0 μg/mL corresponded to 91.5–116% and 99.9%, respectively. The results indicate that the quantification of the concentrations in the range of 0.15 to 2.0 μg/mL was accurate. Conversely, the accuracy values of 0.05 μg/mL were 144–164%, and this exceeded the actual concentration. However, this method can be applied sufficiently to PCB analysis in reclaimed oil given that 0.5 μg/mL denotes the judgment criteria.

### Repeatability and stability

**Fig. 5:**
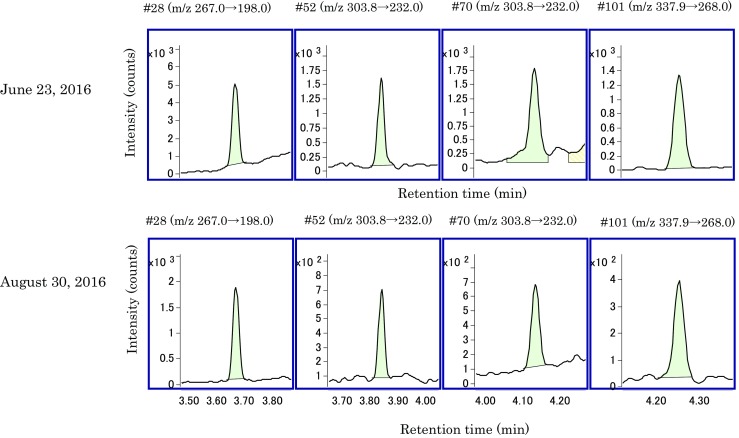
Comparison of SRM chromatograms of IS on June 23, 2016 and August 30, 2016 (#28, 52, 70, 101)

**Fig. 6:**
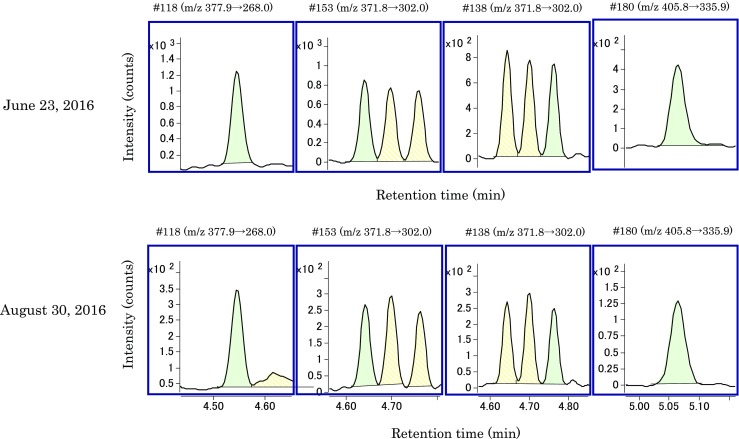
Comparison of SRM chromatograms of IS on June 23, 2016 and August 30, 2016 (#118, 153, 138, 180)

Table 8 shows the relative standard deviation (%RSD) of total PCB concentration (μg/mL) in reclaimed oil containing 0.5 and 0.05 μg/mL KC-mix, and the %RSD values were 3.6 and 5.4%, respectively. This indicates good repeatability even at low concentrations. Figures 5 and 6 show the SRM chromatograms of IS obtained by analysis of the reclaimed oil on June 23, 2016 and on August 30, 2016, respectively. We analyzed 800 or more samples of reclaimed oils between the two analyses. During these 800 or more analyses, a cleaning ion source and an exchanging analysis column were not performed. On the other hand, an exchanging of GC liner and a tuning of quadrupole mass were performed several times. Table 9 shows the signal to noise (S/N) (peak to peak) and the reduction rate ([the S/N of August 30, 2016]/[the S/N of June 23, 2016] × 100 (%)). The reduction rate values of #28, #52, #70, #138, #153, and #180 correspond to 73–107%, and the sensitivity was maintained. The reduction rate values of #101 and #118 are 21 and 20%, respectively. This indicates a reduction in the sensitivity of five chlorinated congeners. Thus, careful quality control is necessary with respect to the sensitivity of components 6, 7, and 8 that corresponds to 5 chlorinated. However, the data shown in “Calibration curve” and “Trueness of total PCBs concentration” were acquired after August 30, 2016 without cleaning ion source and exchanging analysis column. Therefore, this reveals that the proposed method was stable and maintains the required sensitivity even when 800 samples were analyzed.

**Table 8:** %RSD of total PCB concentration in reclaimed oil containing 0.5 and 0.05 μg/mL KC-mix

	1	2	3	4	5	Average	SD	%RSD
0.5 μg/mL	0.533	0.500	0.520	0.490	0.495	0.508	0.0181	3.6
0.05 μg/mL	0.0741	0.0730	0.0818	0.0779	0.0720	0.0758	0.00408	5.4

**Table 9:** Signal to noise (S/N) (peak to peak) on June 23, 2016 and August 30, 2016, and the reduction rate

	#28	#52	#70	#101	#118	#138	#153	#180
June 23, 2016	67.1	25.7	29.1	45.2	40.1	32.5	37.4	28.0
August 30, 2016	48.8	23.8	22.6	9.3	7.9	29.3	31.7	29.9
Reduction rate(%)	73	93	78	21	20	90	85	107

## Conclusion

In this study, a method to analyze PCBs in reclaimed oil was proposed by using fast-GC triple stage quadrupole MS/MS with 13-component quantitation method. The method consists of pretreating 300-fold dilution with hexane, GC/MS/MS analysis of 13.5 min per sample using VF-Rapid MS PCB screen column, and quantitative calculation with 13 components. The method was adopted for the analysis of reclaimed oil containing 0.5 μg/mL PCBs, and this denotes that judgment criteria including accurate quantitation (accuracy value, 94.0–102%) and good repeatability (%RSD, 3.6%) were obtained. Additionally, with respect to the stability of GC/MS/MS system, the required sensitivity was maintained even when 800 samples were analyzed without a cleaning ion source and an exchanging analysis column. These results indicate that the proposed fast and simple analysis method satisfies sensitivity, repeatability, and stability requirements.
